# Association of citrullination with the progression of aortic stenosis

**DOI:** 10.1038/s41598-023-36153-w

**Published:** 2023-06-01

**Authors:** Ah-Ram Kim, Eunhye Ji, Sahmin Lee, Seokchan Hong, Dae-Hee Kim, Jong-Min Song, Duk-Hyun Kang, Jae-Kwan Song

**Affiliations:** 1 Division of Cardiology, Heart Institute, Asan Medical Center, University of Ulsan College of Medicine, Seoul, Republic of Korea; 2 Department of Medical Science, Asan Medical Institute of Convergence Science and Technology, Asan Medical Center, University of Ulsan College of Medicine, 88 Olympic-ro 43 Gil, Songpa-gu, Seoul, 05505 Korea; 3 Division of Rheumatology, Department of Internal Medicine, Asan Medical Center, University of Ulsan College of Medicine, Seoul, Republic of Korea

**Keywords:** Cardiovascular biology, Valvular disease

## Abstract

Despite its clinical importance, biomarkers of disease activity in aortic stenosis (AS) are lacking. We investigated the association between anti-cyclic citrullinated peptide (CCP) antibodies and AS. All 678 patients who underwent echocardiography and anti-CCP antibody testing were analysed. Anti-CCP antibody status was categorized as negative, low-positive, and high-positive. In addition, aortic valve (AV) tissues were obtained from the patients with and without AS to analyze the presence of citrullinated proteins. At baseline, 241 (35.5%) subjects with AV degeneration had a higher rate of anti-CCP antibody positivity (42.7% versus 34.6%, p = 0.035) than those without AV degeneration. Out of the 331 (48.8%) subjects who underwent echocardiographic follow-up, progression of AS was observed in 34 (10.3%) patients, with a higher incidence in the high-positive group compared to the low-positive or negative group (19.0% vs. 11.3% vs. 8.4%, respectively; p = 0.041). On multivariable analysis, high anti-CCP antibody positivity was independently associated with progression to AS (odds ratio: 2.312; 95% confidence interval: 1.006–5.310; p = 0.048). Furthermore, immunohistochemistry and Western blotting revealed increased citrullination in diseased AV compared to normal AV tissue. This study demonstrated that a high positive anti-CCP antibody result is associated with AV degeneration and may be an independent factor for AS progression.

## Introduction

Aortic stenosis (AS) is one of the most common valvular heart diseases in the aging population of developed countries^1^. AS is caused by an inflammatory process resulting from endothelial damage due to mechanical stress and lipid infiltration, leading to leaflet fibrosis, thickening, and calcification^2^. AS typically has a long subclinical period during which it presents as aortic sclerosis with valve calcification, but no significant transvalvular gradient. However, once symptoms develop, the outcome of AS is often fatal. If AS with symptoms is left untreated, the annual mortality rate is approximately 25%, and the median survival is known to be only 2 to 3 years^3^. However, there is currently no effective medical treatment that can delay disease progression and there are no biomarkers of disease activity.

AS shares many pathophysiological similarities with atherosclerosis, including inflammation, calcification, and lipid accumulation^2^. Citrullination is a post-translational modification of proteins that is commonly found in the inflammatory contexts, such as rheumatoid arthritis (RA)^4^. Citrullinated proteins are formed when peptidylarginine is deiminated by the protein-arginine deiminase (PAD) family of enzymes. Citrullination is present in several chronic inflammatory processes and citrullinated proteins have been detected in atherosclerotic plaque^5^. Furthermore, an association between any anti-citrullinated cyclic peptide (CCP) antibody and coronary artery disease (CAD) has been reported^6^, leading to speculation that an immunological mechanism associated with citrullination may contribute to the pathogenesis of AS.

Citrullination during the inflammation appears to stimulate the production of autoantibodies and plasma cells produce anti-citrullinated protein antibodies (ACPA)^7^. Although antibodies exist against several citrullinated proteins and peptides, the most specific and sensitive commercial enzyme-linked immunosorbent assay (ELISA) test for ACPA uses CCPs as a substrate. Therefore, we investigated whether the presence of ACPA, particularly anti-CCP antibodies, could have a potential association with AS and its clinical course. This is a retrospective study of patients who underwent anti-CCP antibody testing, which is commonly used in the differential diagnosis of connective tissue diseases. Despite the large proportion of patients with RA in the study population, we aimed to demonstrate the association between anti-CCP antibody positivity and AS progression independent of underlying connective tissue disease.

## Results

### Presence of citrullinated proteins in human aortic valve

**Figure 1:**
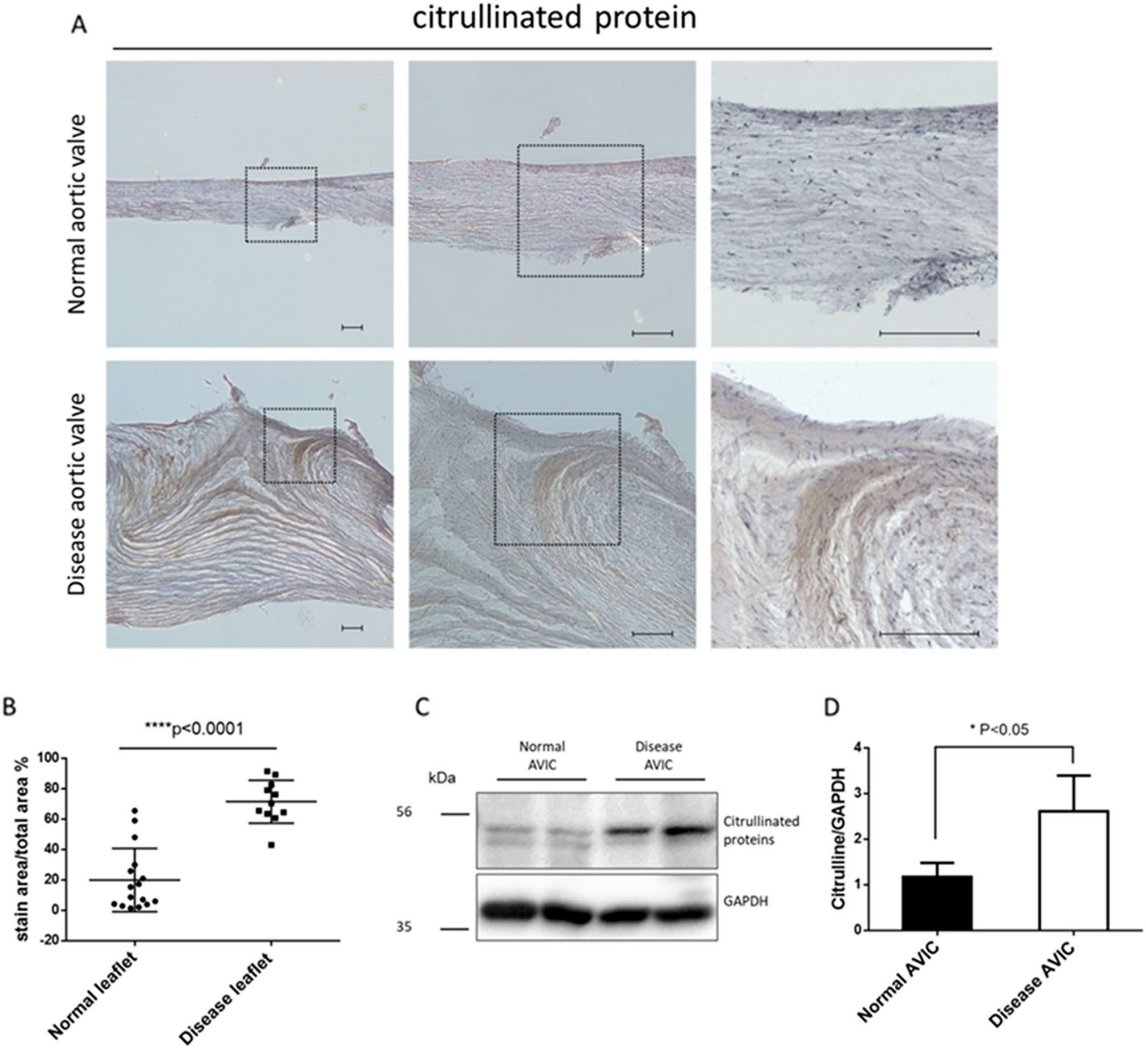
Detection of citrullinated proteins in the aortic valve tissue and aortic valve interstitial cells. (**A**) Immunohistochemistry using citrullinated protein antibody in aortic valve tissue showing more expression of citrullinated protein staining (brown) in disease valve tissue. All scale bar is 200um. The box made of black dashed lines represents the area in the rightmost picture. (**B**) Quantification of staining area in (**A**) tissue. (**C**) Representative Western blots probed for citrullinated proteins also showed a marked expression of citrullinated protein in disease aortic valve interstitial cells. (**D**) Each band was normalized by glyceraldehyde 3-phosphate dehydrogenase (GAPDH). The major band of near 56 k Da in blot (**C**) was identified, which was stronger in disease aortic valve interstitial cells.

We analyzed the presence of citrullinated proteins in aortic valve (AV) tissues and aortic valve interstitial cells (AVICs) by immunohistochemistry and Western blotting. Figure 1 and Supplementary Figs. 1–4 show the presence of prominent citrullinated proteins in the degenerative valve tissues and cells of AS patients, and increased citrullination was observed in areas of diseased AV compared with normal AV tissue. These results suggest a possible link between the citrullinated peptide-related inflammatory process and the pathophysiology of AS.

### Characteristics of study subjects according to aortic valve degenerative changes

After eligibility screening, a total of 678 patients with both anti-CCP antibody and transthoracic echocardiography results were included (Supplementary Fig. 5). Among the 678 patients, 254 had a positive anti-CCP result and 424 had a negative anti-CCP result. In addition, 241 patients had degenerative changes in the AV and the other 437 patients had normal AV morphology. Table 1 shows the baseline characteristics categorized by degenerative changes in AV. In general, patients with degenerative AV were older and had higher rates of combined hypertension, diabetes, and coronary artery disease (CAD). Patients with degenerative AV had a higher rate of anti-CCP positivity compared to patients with normal AV (42.7% vs. 34.6%, p = 0.035). The combined rheumatoid arthritis (RA) rate was higher in the degenerative AV group (42.7% vs. 27.9%, p < 0.001). Peak aortic jet velocity was higher in the degenerative AV group (Table 1).

**Table 1:** Clinical characteristics of patients according to the aortic valve morphology.

Characteristics	Total (n = 678)	Degenerative change (N = 241)	Normal morphology (n = 437)	p value
Age (years), mean ± SD	62.1 ± 13.8	69.8 ± 9.4	57.9 ± 14.0	< 0.001
Male, *n* (%)	288 (42.5)	106 (44.0)	182 (41.6)	0.556
BMI (kg/m^2^), mean ± SD	23.2 ± 3.9	23.2 ± 3.9	23.2 ± 4.0	0.846
Anti-CCP Ab positive, *n* (%)	254 (37.5)	103 (42.7)	151 (34.6)	0.035
Hypertension, *n* (%)	204 (30.1)	98 (40.7)	106 (24.3)	< 0.001
Diabetes mellitus, *n* (%)	141 (20.8)	62 (25.7)	79 (18.1)	0.019
Coronary artery disease, *n* (%)	82 (12.1)	46 (19.1)	36 (8.2)	< 0.001
Chronic kidney disease, *n* (%)	62 (9.1)	26 (10.8)	36 (8.2)	0.270
Cerebrovascualr accident, *n* (%)	24 (3.5)	12 (5.0)	12 (2.7)	0.132
Atrial fibrillation/flutter, *n* (%)	50 (7.4)	24 (10.0)	26 (5.9)	0.056
Rheumatoid arthritis, *n* (%)	225 (33.2)	103 (42.7)	122 (27.9)	< 0.001
Interstitial lung disease, *n* (%)	161 (23.7)	50 (20.7)	111 (25.4)	0.173
Current or past smoker, *n* (%)	208 (30.7)	77 (32.0)	131 (30.0)	0.594
Baseline AV V_max_ (m/sec), mean ± SD	1.56 ± 0.71	1.91 ± 1.03	1.37 ± 0.30	< 0.001
Baseline LVEF (%), mean ± SD	58.7 ± 10.9	58.7 ± 10.4	58.8 ± 11.1	0.937
Combined other valve disease, *n* (%)	78 (11.5)	32 (13.3)	46 (10.5)	0.282

*SD* standard deviation, *BMI* body mass index, *Anti*-*CCP Ab* anti-cyclic citrullinated peptide antibody, *AV V*_max_ peak aortic jet velocity, *LVEF* left ventricle ejection fraction.

### Clinical outcomes and anti-CCP antibody results

**Figure 2:**
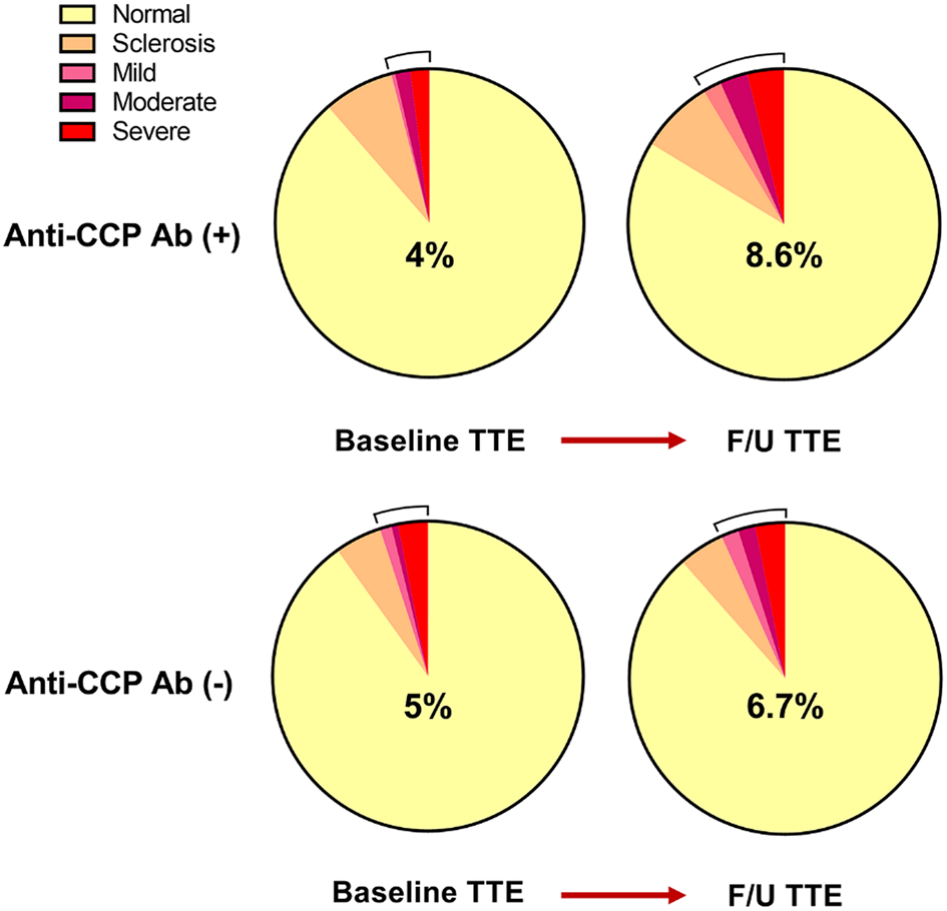
Changes in aortic stenosis severity according to anti-CCP antibody positivity. In the anti-CCP antibody-positive group, the proportion of at least mild aortic stenosis increased from 4.0 to 8.6% on last follow-up echocardiography. The proportion increased from 5 to 6.7% in the negative group, which is less than that in the positive group.

Follow-up echocardiography was performed after more than one year in 331 patients, and the median follow-up was 4.6 years (interquartile range (IQR): 2.0–8.0 years). Supplementary Fig. 6 shows the changes in AS severity from baseline to follow-up echocardiography. AS progression, the primary outcome, was defined when the severity of AS worsened by at least one grade at the subsequent echocardiographic evaluation and was observed in 34 (10.3%) patients. Patient characteristics based on progression of AS on follow-up echocardiography are shown in Table 2. The group with AS progression was significantly older and had more combined CAD and higher baseline AV Vmax. Although the rate of anti-CCP antibody positivity was higher in the AS progression group, the difference was not statistically significant (44.1% vs. 30.0%, p = 0.092). The absolute anti-CCP antibody titre was 88.4 ± 130.3 U/mL in the AS progressed group and 52.3 ± 104.6 U/mL in the AS not progressed group. There were no significant differences in C-reactive protein (CRP) and erythrocyte sedimentation rate (ESR) between the two groups. Figure 2 shows the changes in AS severity according to the anti-CCP antibody test result. The proportion of at least mild AS increased more in the anti-CCP-positive group, from 4.0 to 8.6%, than in the anti-CCP-negative group, which only changed from 5.0 to 6.7%.

**Table 2:** Characteristics by the progression of aortic stenosis.

Characteristics	AS progressed (N = 34)	AS not progressed (N = 297)	p value
Age (years), mean ± SD	66.7 ± 7.8	60.1 ± 12.5	< 0.001
Male, *n* (%)	16 (47.1)	133 (44.8)	0.800
BMI (kg/m^2^), mean ± SD	24.5 ± 3.0	23.3 ± 3.7	0.090
Anti-CCP Ab positive, *n* (%)	15 (44.1)	89 (30.0)	0.092
Anti-CCP Ab titre (U/mL), mean ± SD	88.4 ± 130.3	52.3 ± 104.6	0.127
Hypertension, *n* (%)	10 (29.4)	80 (26.9)	0.759
Diabetes mellitus, *n* (%)	6 (17.6)	57 (19.2)	0.828
Coronary artery disease, *n* (%)	12 (35.3)	36 (12.1)	< 0.001
Chronic kidney disease, *n* (%)	3 (8.8)	29 (9.8)	0.999
Cerebrovascular accident, *n* (%)	0 (0.0)	4 (1.3)	0.999
Atrial fibrillation/flutter, *n* (%)	5 (14.7)	24 (8.1)	0.200
Rheumatoid arthritis, *n* (%)	11 (32.4)	63 (21.2)	0.140
Interstitial lung disease, *n* (%)	9 (26.5)	78 (26.3)	0.979
Current or past smoker, *n* (%)	13 (38.2)	97 (32.7)	0.513
Baseline AV V_max_ (m/sec), mean ± SD	1.84 ± 0.49	1.39 ± 0.36	< 0.001
Baseline LVEF (%), mean ± SD	61.0 ± 7.1	58.7 ± 10.8	0.229
Combined other valve disease, *n* (%)	10 (29.4)	50 (16.8)	0.071
Last f/u AV V_max_ (m/sec), mean ± SD	3.14 ± 0.98	1.42 ± 0.35	< 0.001
CRP (mg/dL), median (IQR)	2.3 (0.5–5.2)	1.6 (0.4–6.3)	0.668
ESR (mm/hr), median (IQR)	55.0 (35.8–83.5)	43.0 (20.5–71.5)	0.108

*SD* standard deviation, *BMI* body mass index, *Anti*-*CCP Ab* anti-cyclic citrullinated peptide antibody, *AV V*_max_ peak aortic jet velocity, *LVEF* left ventricle ejection fraction, *CRP* C-reactive protein, *IQR* interquartile range, *ESR* erythrocyte sedimentation rate.

The anti-CCP antibody-positive group was further divided into two groups: low-positive, with levels higher than the upper limit of normal (ULN) but less than the median (322.0 U/mL for fluorescence enzyme immunoassay (FEIA) and 200.0 U/mL for chemiluminescent microparticle immunoassay (CMIA), respectively), and high-positive, with levels greater than or equal to the median. Characteristics categorized according to the anti-CCP antibody titre are shown in Table 3. Compared with the negative group, the high-positive group was older and had higher rates of combined hypertension, RA, and interstitial lung disease (ILD). Although the rates of male proportion, smoking, combined CAD, and chronic kidney disease were lower in the low-positive and high-positive groups compared with the negative group, the positive groups had a higher frequency of degenerative changes of the AV with an increasing trend (p = 0.042). There were no significant differences in CRP and ESR between the groups.

**Table 3:** Characteristics according to the anti-CCP titre group.

	Negative (N = 424)	Low positive (N = 138)	High positive (N = 116)	p value
Age (years), mean ± SD	59.4 ± 14.9	65.6 ± 10.4	67.8 ± 10.1	< 0.001
Male, *n* (%)	206 (48.6)	42 (30.4)	40 (34.5)	< 0.001
BMI (kg/m^2^), mean ± SD	23.2 ± 3.9	23.7 ± 3.9	22.4 ± 3.9	0.022
Hypertension, *n* (%)	113 (26.7)	48 (34.8)	43 (37.1)	0.014
Diabetes mellitus, *n* (%)	94 (22.2)	24 (17.4)	23 (19.8)	0.392
Coronary artery disease, *n* (%)	60 (14.2)	15 (10.9)	7 (6.0)	0.016
Chronic kidney disease, *n* (%)	50 (11.8)	5 (3.6)	7 (6.0)	0.010
Cerebrovascular accident, *n* (%)	17 (4.0)	3 (2.2)	4 (3.4)	0.571
Atrial fibrillation/flutter, *n* (%)	35 (8.3)	8 (5.8)	7 (6.0)	0.312
Rheumatoid arthritis, *n* (%)	55 (13.0)	85 (61.6)	85 (73.3)	< 0.001
Interstitial lung disease, *n* (%)	86 (20.3)	41 (29.7)	34 (29.3)	0.013
Current or past smoker, *n* (%)	144 (34.0)	34 (24.6)	30 (25.9)	0.035
Baseline AV V_max_ (m/s), mean ± SD	1.54 ± 0.72	1.55 ± 0.67	1.65 ± 0.70	0.279
Baseline LVEF (%), mean ± SD	57.6 ± 12.2	60.8 ± 7.3	60.5 ± 8.4	0.002
Combined other valve disease, *n* (%)	65 (15.3)	7 (5.1)	6 (5.2)	< 0.001
AV degeneration at baseline, *n* (%)	138 (32.5)	55 (39.9)	48 (41.4)	0.042
Anti-CCP Ab titre (U/mL), mean ± SD	0.8 ± 1.9	87.9 ± 79.5	284.4 ± 68.0	< 0.001
CRP (mg/dL), median (IQR)	2.8 (0.5–8.4)	1.6 (0.4–5.5)	1.7 (0.5–5.8)	0.055
ESR (mm/hr), median (IQR)	42.5 (19.0–73.0)	43.0 (22.0–76.0)	52.0 (26.0–87.0)	0.143

*SD* standard deviation, *BMI* body mass index, *AV V*_max_ peak aortic jet velocity, *LVEF* left ventricle ejection fraction, *AV* aortic valve, *Anti*-*CCP Ab* anti-cyclic citrullinated peptide antibody, *CRP* C-reactive protein, *IQR* interquartile range, *ESR* erythrocyte sedimentation rate.

**Figure 3:**
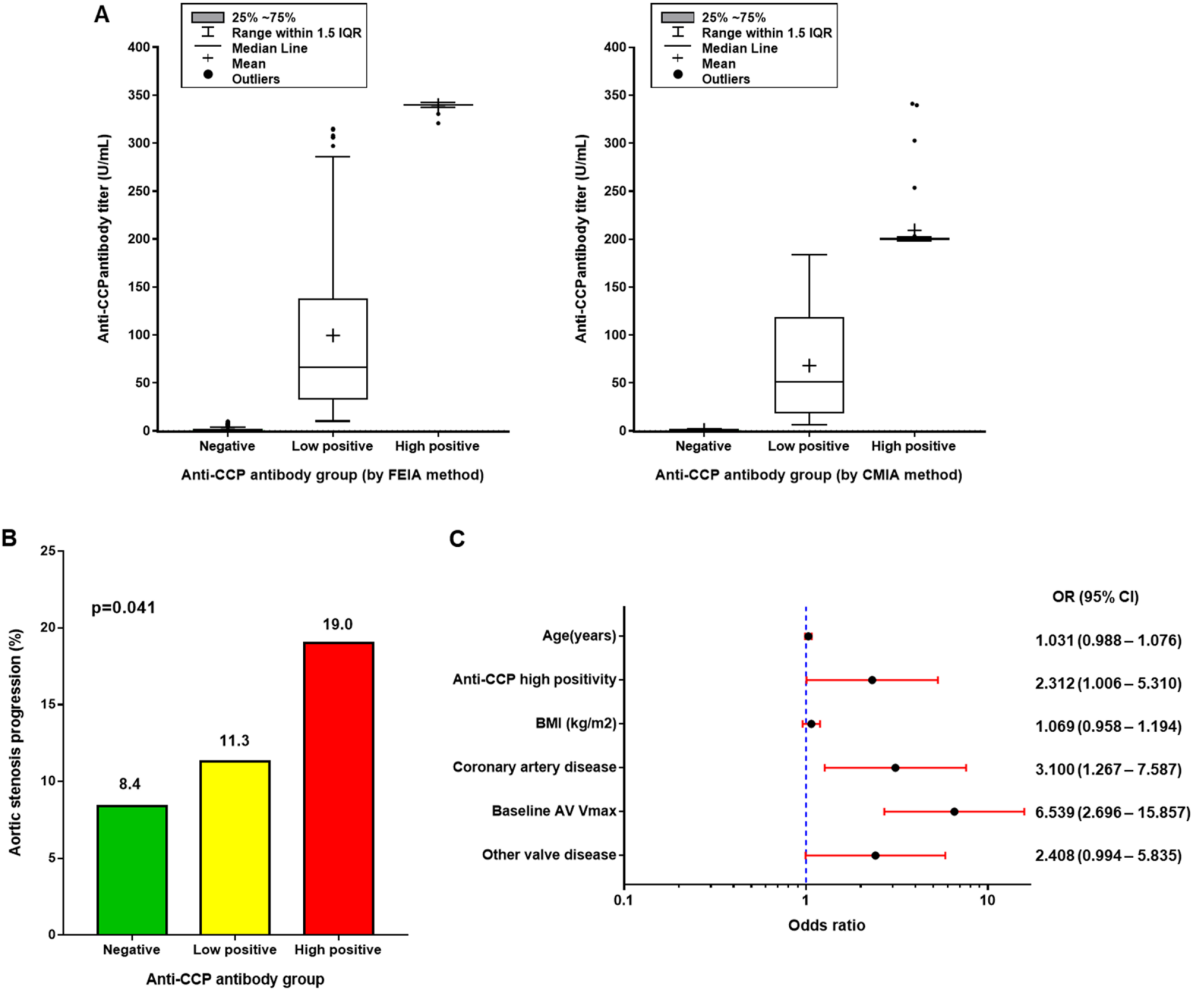
Anti-CCP antibody titre groups and clinical outcomes. (**A**) Graphs illustrating anti-CCP antibody titre in the three groups divided by anti-CCP antibody level of each method. (**B**) Bar graphs showing the percentage of aortic stenosis progression on follow-up echocardiography. (**C**) Factors associated with the progression of aortic stenosis.

Table 4 shows the clinical outcomes categorized by the anti-CCP antibody level. The median clinical follow-up of all patients was 3.5 years (IQR: 1.1–7.5 years), and the median clinical follow-up of patients with echocardiographic follow-up was 6.2 years (IQR: 3.4–9.8 years). Progression of AS occurred more frequently in the high-positive group than in the low-positive or negative groups (19.0% vs. 11.3% vs. 8.4%, respectively; p = 0.041). The rates of all-cause mortality, cardiovascular death, aortic valve replacement (AVR), and the composite of all-cause death and AVR were not statistically different between the groups. Figure 3 summarizes the anti-CCP antibody titre and the proportion of AS progression among three different anti-CCP antibody titre groups.

**Table 4:** Clinical outcomes according to the anti-CCP antibody titre.

	Negative (N = 424)	Low positive (N = 138)	High positive (N = 116)	p value
Primary outcome				
AS progression*	19/227 (8.4)	7/62 (11.3)	8/42 (19.0)	0.041
Secondary outcomes				
Composite of death, AVR, *n* (%)	180 (42.5)	65 (47.1)	46 (39.7)	0.855
All-cause mortality, *n* (%)	171 (40.3)	62 (44.9)	44 (37.9)	0.906
CV mortality, *n* (%)	14 (3.3)	4 (2.9)	4 (3.4)	0.822
AVR, *n* (%)	20 (4.7)	6 (4.3)	5 (4.3)	0.826

Values are *n*/total number (%).

*AS* aortic stenosis, *AVR* aortic valve replacement, *CV* cardiovascular.

*Evaluated only in patients whose follow-up echocardiographic data were available.

### Association between anti-CCP antibody levels and progression of aortic stenosis

Univariate and multivariate analysis were performed to identify the factors influencing the progression of AS. Multivariable analysis showed that anti-CCP-high positivity was a significant factor for AS progression (adjusted odds ratio [OR], 2.312; 95% CI 1.006–5.310; p = 0.048, Fig. 3C and Table 5). Also, combined CAD and baseline AV Vmax were independent associated factors for AS progression (adjusted OR, 3.100; 95% CI 1.267–7.587; p = 0.013, and adjusted OR 6.539; 95% CI 2.696–15.857; p < 0.001, respectively). Of the 331 patients who underwent follow-up echocardiography, follow-up anti-CCP antibody results were available for 127 patients, and the proportion of high-positive results in the AS progression group remained unchanged during the follow-up period (Supplementary Fig. 7).

**Table 5:** Factors associated with the progression of aortic stenosis.

	Univariate analysis			Multivariable analysis		
	OR	95% CI	P value	Adjusted OR	95% CI	P value
Age (years)	1.056	1.018–1.095	0.004	1.031	0.988–1.076	0.157
Anti-CCP high positivity	1.932	0.931–4.008	0.077	2.312	1.006–5.310	0.048
BMI (kg/m^2^)	1.087	0.987–1.197	0.091	1.069	0.958–1.194	0.234
Coronary artery disease	3.955	1.804–8.670	0.001	3.100	1.267–7.587	0.013
Baseline AV V_max_ (m/s)	8.994	3.713–21.786	< 0.001	6.539	2.696–15.857	< 0.001
Other valve disease	2.058	0.927–4.571	0.076	2.408	0.994–5.835	0.052

*OR* odds ratio, *CI* confidence interval, *Anti*-*CCP* anti-cyclic citrullinated peptide, *BMI* body mass index, *AV V*_max_ peak aortic jet velocity.

## Discussion

In the present study, we investigated the association between AS and citrullination expressed as anti-CCP antibodies. Our study had several significant findings: (i) diseased AV showed increased citrullination compared with normal AV; (ii) the anti-CCP positive rate was higher in the patients with degenerative AV; (iii) the high anti-CCP antibody level was found to be independently associated with the progression of AS. To our knowledge, these findings are the first evidence of an association between AS and citrullination.

Citrullination has been reported as a component of several chronic inflammatory processes, and increased citrullination is known to be an inflammation-dependent process^8^. Anti-citrullinated peptide antibodies are commonly found in the serum of patients with RA^9^, and elevated levels of anti-CCP antibodies in a healthy population suggest a high risk of developing RA^10^. In this study, we included a population without autoimmune disease and were able to compare AS progression rates according to anti-CCP antibody titre group and combined connective tissue disease status. Supplementary Fig. 8 shows an increasing trend of AS progression with anti-CCP antibody positivity in both subgroups with and without combined RA/ILD disease, although statistically insignificant. There was no significant interaction between the combined RA/ILD and anti-CCP titre group with respect to AS progression (P_interaction_ = 0.778). We also demonstrated the presence of citrullinated proteins in human aortic valve biopsies obtained from patients without clinical evidence of connective tissue disease. In the valves of AS patients, marked citrullinated proteins were visualized compared to normal valve tissue. Despite its limited strength, the citrullinated protein-related inflammatory process may play a role in the pathophysiology of AS, even in patients without underlying connective tissue disease.

Citrullination is a post-translational modification mechanism mediated by PAD enzymes. The expression of PAD enzymes and the significant deposition of citrullinated protein in atherosclerotic plaques suggest that the increased expression of citrullinated protein is mediated by the activity of PAD enzymes in inflammatory diseases, including AS. We confirmed the expression of the PAD2 gene, which belongs to the family of PAD enzymes, in hAVICs isolated from the AS patients using PCR experiments (Supplementary Fig. 9). While the citrullination process is indeed dependent on the activity of PAD, it is crucial to identify the specific proteins that PAD targets for citrullination in order to fully understand the mechanisms of action. Interestingly, a major band at approximately 56 kDa appeared in the Western blot using the anti-citrulline antibody (Supplementary Fig. 1), whereas a strongly detected band at approximately 45 kDa appeared in the results using the anti-modified citrulline antibody (Supplementary Fig. 3). When the amount of protein was increased to compare the results of both antibodies on the same sample, multiple bands of different sizes were observed, and the pattern of bands detected was similar regardless of the type of antibody, despite a difference in band density (Supplementary Fig. 4). Considering that a previous study has shown that citrullinated fibrinogen and citrullinated vimentin are associated with increased aortic plaque^5^, and both proteins can be detected in the same region around the 56 kDa marker, we can propose fibrinogen and vimentin as candidates of the main target protein in the citrullination process related to the pathophysiology of AS. We also confirmed the presence of citrullinated forms of a-enolase located near 47 kDa, as well as fibrinogen and vimentin (Supplementary Fig. 10), and there may be other candidate proteins that have not been discovered and require further research. The protein identification of the main band detected may be a critical step in understanding the novel pathophysiological mechanisms of AS and may have a significant impact on the research of target discovery for the development of AS treatment.

In this study, citrullination was evaluated as measured by anti-CCP antibody levels. However, citrullination and the formation of citrullinated protein-related antibodies are different processes. It is not currently possible to measure the direct activity of citrullination or citrullinated protein from serum in clinical practice. Although there are other antibodies to citrullinated peptides or proteins besides anti-CCP, such as fibrin, vimentin, filaggrin, and keratin^11^, the anti-CCP antibody test is the only currently available and widely used commercial test for ACPA. Despite this limitation that we used anti-CCP antibody to evaluate the citrullination and related antibody-mediated reaction, we found that anti-CCP antibody positivity was associated with the AV degeneration and the progression of AS. In addition, our study found increased citrullination in diseased AV tissue in patients without RA, which is an important finding suggesting that increased citrullination may contribute to the pathophysiology of AS even in patients without clinical RA. Furthermore, elevated levels of anti-CCP antibodies may in part reflect increased citrullination and thus be predictive of the progression of AS.

On the other hand, the level of circulating antibodies may not accurately reflect the inflammatory activity of the tissue. In the present study, calcified AV tissue showed increased citrullination in laboratory tests. Further studies should analyze tissue inflammatory activity and serologic tests that measure ACPA, including anti-CCP antibodies and antibodies to other peptides/proteins.

In this study, anti-CCP antibody positivity was associated with progression of AS but not with AVR or mortality. This finding may be attributed to the characteristics of the subjects in this study. Compared with the general population, the incidence of combined ILD was higher in this cohort^12,13^. Most of the patients with ILD succumbed to the progression of ILD or combined pneumonia. Therefore, ILD may have had a greater impact on clinical outcomes than the progression of AS in these patients. All-cause mortality and the rate of combined ILD were highest in the low-positive group, while cardiovascular death was lowest. These findings also suggest that ILD may have had a greater impact on the clinical course of these patients than anti-CCP antibody positivity, and that they may have died of an ILD-related complication prior to AVR even with advanced AS.

As previously described, atherosclerosis and calcific AS share common risk factors and show many similarities in pathophysiology^2,14^. However, the presence of combined CAD and the anti-CCP positivity were independent factors for the progression of AS in this study. Furthermore, the male proportion, smoking, combined CAD, and chronic kidney disease rates were lower in the anti-CCP positive groups, especially in the high-positive group. These findings suggest that increased citrullination and its associated inflammatory response may play an independent role in the pathogenesis of AS, in addition to the known risk factors such as age, hypertension, diabetes, and smoking^15,16^. Therefore, anti-CCP antibodies may be a novel biomarker to predict the presence or progression of AS.

At baseline echocardiography, patients with AV degenerative changes had a higher rate of combined RA. Since the production of anti-CCP antibody is known to be specific for RA^9^, patients with RA, especially those seropositive for anti-CCP antibody, may require echocardiographic evaluation of the AV, and patients with high anti-CCP antibody titres and significant AS at baseline may need to be followed closely for progression of AS.

This study has several limitations. First, the design was retrospective, which means that data collection was not systematic. Therefore, the results should be considered as hypothesis-generating. Second, the most important limitation of this study is the selection for the study group. The cohort was formed by identifying individuals who underwent anti-CCP antibody testing, which was usually done in cases of clinical suspicion of connective tissue disease. As a result, the study included a large proportion of patients with RA and/or ILD, which accounted for 47.9% of the study population. This limited cohort, which included only patients who had undergone at least one echocardiogram and anti-CCP antibody testing, may also introduce further selection bias. Although the results suggest that there is an association between anti-CCP antibody positivity and AS progression independent of the presence of combined connective tissue disease, we should be cautious in interpreting the results of this study, and further research is needed to extrapolate them to the general population. Third, although serial echocardiographic data were available for more than half of the total population, the follow-up interval varied widely among subjects. It was difficult to analyze clinical outcomes over the same time interval because of the limitations of the retrospective design. Finally, the study was conducted in a single centre and further large studies are warranted.

In conclusion, the presence of anti-CCP antibody, especially at high titre, was associated with degenerative changes of the AV at baseline echocardiography. Furthermore, anti-CCP antibody positivity was identified as an independent factor in the progression of AS. These results suggest that citrullination may play a role in the pathogenesis of AS. In addition, anti-CCP antibodies may serve as a biomarker to predict the progression of AS. Further extensive research, including laboratory studies, is needed to confirm these findings.

## Methods

### Human aortic valve tissue

AV tissues were obtained from patients with or without AS at the time of surgical AVR or heart transplantation. We obtained diseased valve tissue from 11 patients who underwent AVR surgery and normal valve tissue from 8 patients who underwent heart transplantation surgery. None of the subjects had any combined connective tissue diseases, such as ILD or RA. This study adhered to the Declaration of Helsinki, and the Institutional Review Board (IRB) of Asan Medical Center (Seoul, Korea) approved the study protocol for human specimen collection (IRB No.: 2017-0556), and all patients provided written informed consent.

### Isolation of primary aortic valve interstitial cells

One leaflet of the obtained human AV was cut into 4 or 5 pieces and digested with collagenase mixture at 37 °C for 30 min to remove AV endothelial cells. Then, AVICs were obtained by additional incubation for 1.5 h at 37 °C. Isolated human AVICs were cultured in Dulbecco’s modified Eagle’s medium/nutrient mixture F-12 (DMEM/F12) supplemented with 1% antibiotic–antimycotic and 10% fetal bovine serum (FBS). Only passages 2–5 were used for Western blot.

### Western blot

Harvested valves and isolated AVICs were homogenized in 1X radioimmunoprecipitation assay (RIPA) with proteinase inhibitors and lysates were loaded on sodium dodecyl sulfate–polyacrylamide gel electrophoresis (SDS-PAGE). Separated proteins were transferred to a polyvinylidene difluoride (PVDF) membrane, which was then blocked with 5% bovine serum albumin (BSA) for 1 h at room temperature. They were then detected with pan-citrulline antibody (ab6464, 1:1000, Abcam, Cambridge, UK), anti-modified citrulline antibody(MABS487, 1:1000, Merck Millipore, Massachusetts, USA), anti-citrullinated vimentin antibody(22054, 1:1000, Cayman chemical, Michigan, USA), anti-citrullinated fibrinogen antibody(17088, 1:1000, Cayman Chemical, Michigan, USA), anti-citrullinated α-enolase antibody(23000, 1:1000, Cayman Chemical, Michigan, USA), peroxidase affiniPure goat anti-human IgG(109-035-008, 1:5000, Jackson ImmunoResearch, Pennsylvania, USA), and horseradish peroxidase (HRP)-conjugated anti-rabbit IgG antibody.

### Immunohistochemistry

The frozen block obtained from the tissue fixation and sucrose descent step was sectioned at 8 µm thickness using a microtome. The sectioned tissues were washed with tris-buffered saline (TBS) containing 0.025% Triton x-100 and incubated in blocking solution (code X0909, Dako, Hovedstaden, Denmark) for 30 min at room temperature. After three washes, primary antibodies (ab6464, 1:1000, Abcam, Cambridge, UK and MABS487, 1:1000, Merck Millipore, Massachusetts, USA) were applied to the tissue slides overnight at 4 °C, while the negative control was treated with TBS. The tissues were then rinsed with washing buffer twice for 5 min each and incubated in peroxidase blocking solution at room temperature (RT) for 10 min to quench endogenous peroxidase activity. After two more washes, the tissues were incubated with rabbit/mouse secondary antibody from the Dako REAL Envision detection system (K5077, Dako, Hovedstaden, Denmark) for 30 min at RT. After washing off the antibody solution, the tissues were developed with a mixture of diaminobenzidine (DAB) + chromogen and substrate buffer (K5077, Dako, Hovedstaden, Denmark) for 10 min. The tissues were then immersed in water to stop the reaction and stained with hematoxylin for 2 min. The tissues were then dehydrated, coverslipped, and examined under a microscope.

### Study population

This study was conducted at Asan Medical Center, a tertiary referral hospital in Seoul, South Korea, and was a retrospective observational study. Subjects aged ≥ 18 years who underwent both echocardiography and anti-CCP antibody testing between April 2004 and July 2020 were enrolled. The analysis included data from transthoracic echocardiography which was performed within 6 months before or after the anti-CCP antibody test. Subjects who met any of the following criteria were excluded from the study: (i) those who underwent AV surgery or intervention prior to the anti-CCP antibody test, (ii) those with other significant valvular heart disease (greater than moderate severity), (iii) those with rheumatic valve disease, and (iv) those with bicuspid AV.

The study protocol was approved by the IRB of the Asan Medical Center (IRB No.:2019-1024). Due to the retrospective nature of the study, the committee waived the requirement for informed consent.

The patients’ medical records were reviewed for demographic details, clinical diagnosis, and other laboratory data. ESR and plasma CRP measurements were obtained in addition to anti-CCP antibody titres. Disease severity and AV morphology were assessed by two-dimensional echocardiography and Doppler measurements such as peak trans-aortic velocity. There were no cases of missing clinical data.

### Echocardiographic evaluation

All patients (N = 678) underwent echocardiography with M-mode, two-dimensional and color Doppler by an experienced echocardiographer. AV degeneration was defined as thickening of the AV leaflet or calcification involving more than one leaflet. AS severity was graded as none, mild, moderate, or severe according to the recommendations of the American College of Cardiology/American Heart Association and the European Society of Cardiology, integrating structural, Doppler, and quantitative parameters^17,18^. The biplane Simpson volumetric method, combining apical 4- and 2-chamber views, was used to measure left ventricular ejection fraction^19^. Echocardiographic data for at least 12 months were available for 331 (48.8%) of the total subjects. For these subjects, the progression of AS was assessed.

### Anti-CCP antibody assay

Anti-CCP antibodies were detected by the FEIA method (ELIA™, Phadia, Uppsala, Sweden) or the CMIA method (Architect™, Abbott Laboratories, Illinois, USA). In our center, these two methods were used in different periods: the FEIA method was used from 2004 to 2018, while the CMIA method was used from 2012 to the present. These two methods had previously shown comparable diagnostic performance^20^. Anti-CCP status was considered positive if the concentration was > 10 U/mL (by FEIA) or ≥ 5.0 U/mL (by CMIA). If the value was less than the ULN, it was considered negative. To compare the values obtained by the FEIA and CMIA methods, the titres were divided into three categories as follows: negative (less than the ULN), low-positive (between the ULN and the median value of 322.0 U/mL for FEIA or 200.0 U/mL for CMIA), and high-positive (equal to or greater than the median).

### Study outcomes

Progression of AS, defined as an increase in AS severity of at least one grade at subsequent echocardiographic evaluation, was assessed as the primary outcome. Secondary outcomes included the rate of AVR, all-cause mortality, and cardiovascular-cause death. AVR included both surgical and transcatheter AV replacement.

### Statistical analysis

Continuous variables are presented as means ± standard deviations or medians (IQR), whereas categorical variables are presented as counts (%). Student *t*-tests or Mann–Whitney *U* tests were used to compare continuous variables, and chi-squared or Fisher’s exact tests were used to compare categorical variables. Analysis of variance (ANOVA) and chi-squared test for trend were used to assess differences between anti-CCP antibody titre groups. Binary logistic regression analysis was used to perform univariate analyses for each variable to examine the association between measured variables and AS progression. Stepwise multivariable logistic regression analysis was used to determine independent factors of AS progression, including variables with a *P* value < 0.10 in univariate analysis. All tests were two-tailed, and *P* values less than 0.05 were considered statistically significant. Statistical analyses were performed with the Statistical Package for the Social Sciences, version 22.0 (IBM Corp., Armonk, NY, USA).

## Supplementary Information

Supplementary Figures.

## Data Availability

The data that support the findings of this study are available from the corresponding author upon reasonable request.
