# Supplementary material for: Association of citrullination with the progression of aortic stenosis
Source: Sci Rep. 2023 Jun 1;13:8919. doi: 10.1038/s41598-023-36153-w (PMC10235071; doi:10.1038/s41598-023-36153-w)
Supplement: Supplementary file 1 — Supplementary Figures. [file 41598_2023_36153_MOESM1_ESM.docx]

**SUPPLEMENTARY FIGURE**

**Supplementary Figure 1.** **Detection of citrullinated proteins in the aortic valve interstitial cells using anti-citrulline antibodies**

The representative Western blots probed for citrullinated proteins showed a significant expression of citrullinated proteins in disease aortic valve interstitial cells. This is the full-length original membrane data of Figure 1C.

**Supplementary Figure 2. Immunohistochemistry using anti-modified citrulline antibody**

Immunohistochemistry results using anti-modified citrulline antibody staining on slides of normal aortic valve tissue from a heart transplant patient and disease aortic valve tissue from a patient who underwent aortic valve replacement surgery.

**Supplementary Figure 3. Western blotting using anti-modified citrulline antibody**


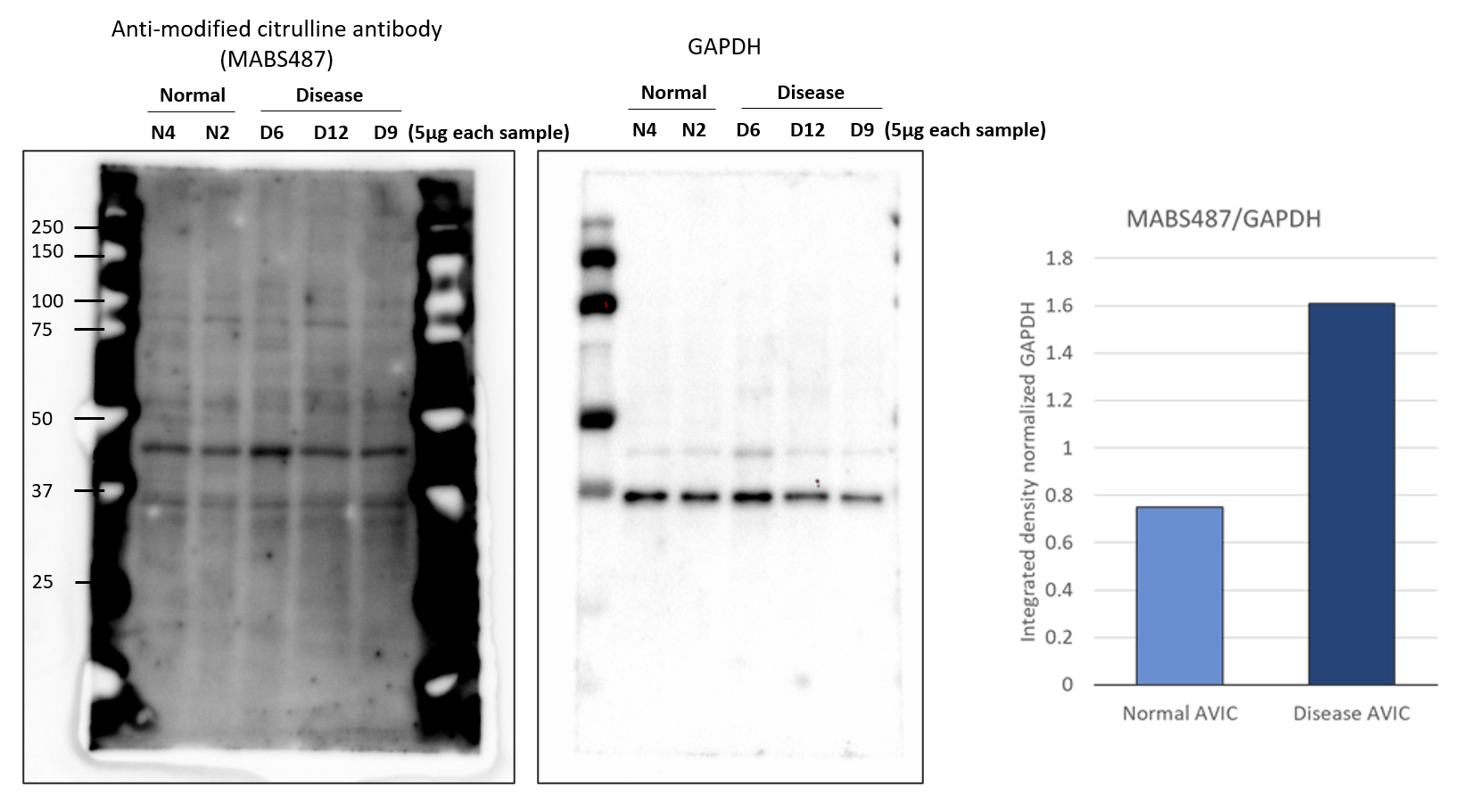


Representative image of Western blot and quantification graphs of bands sized at 45kD using anti-modified citrulline antibody in normal aortic valve interstitial cells (AVICs) which were isolated from the patients who received heart transplantation and in disease AVICs isolated from the AS patients who received aortic valve replacement.

**Supplementary Figure 4. Detection patterns of anti-modified citrulline antibody and anti-citrulline antibody**

Comparison of detection patterns of anti-modified citrulline antibody (MABS487) and anti-citrulline antibody (ab6464) in AVICs isolated from aortic valve tissue.

**Supplementary Figure 5. Study flow chart**

**
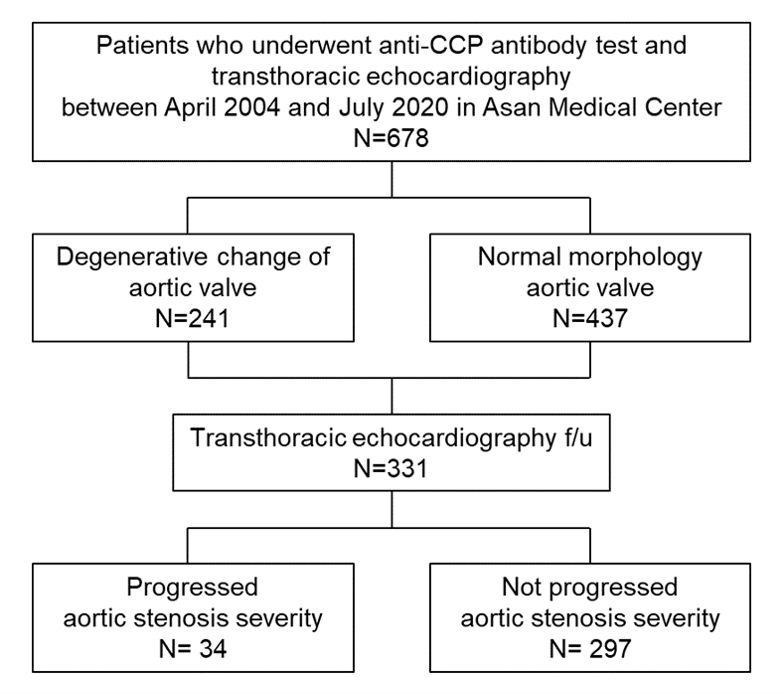
**

A total of 678 subjects underwent both transthoracic echocardiography and anti-CCP antibody test. Follow-up transthoracic echocardiography was performed in 331 subjects.

**Supplementary Figure 6. Changes in aortic stenosis severity on follow-up echocardiography**

**
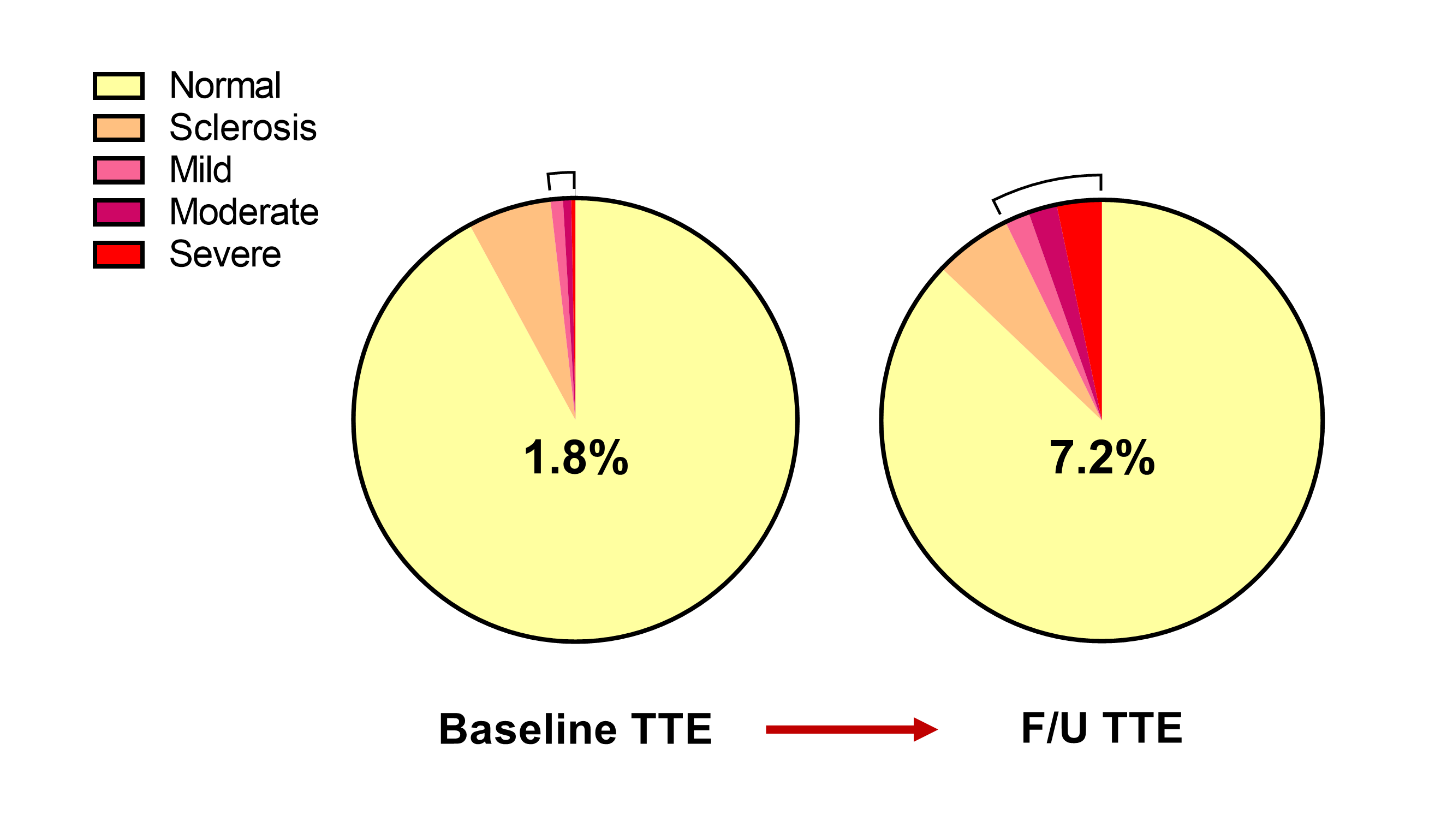
**

The follow-up echocardiography data were available for 331 out of total 678 patients. The proportion of aortic stenosis with more than mild severity was 1.8% on baseline echocardiography and increased to 7.2% on last follow-up echocardiography. The echocardiography was followed up for a median of 4.6 years [IQR: 2.0 – 8.0].

**Supplementary Figure 7. Changes in the anti-CCP antibody titres group**


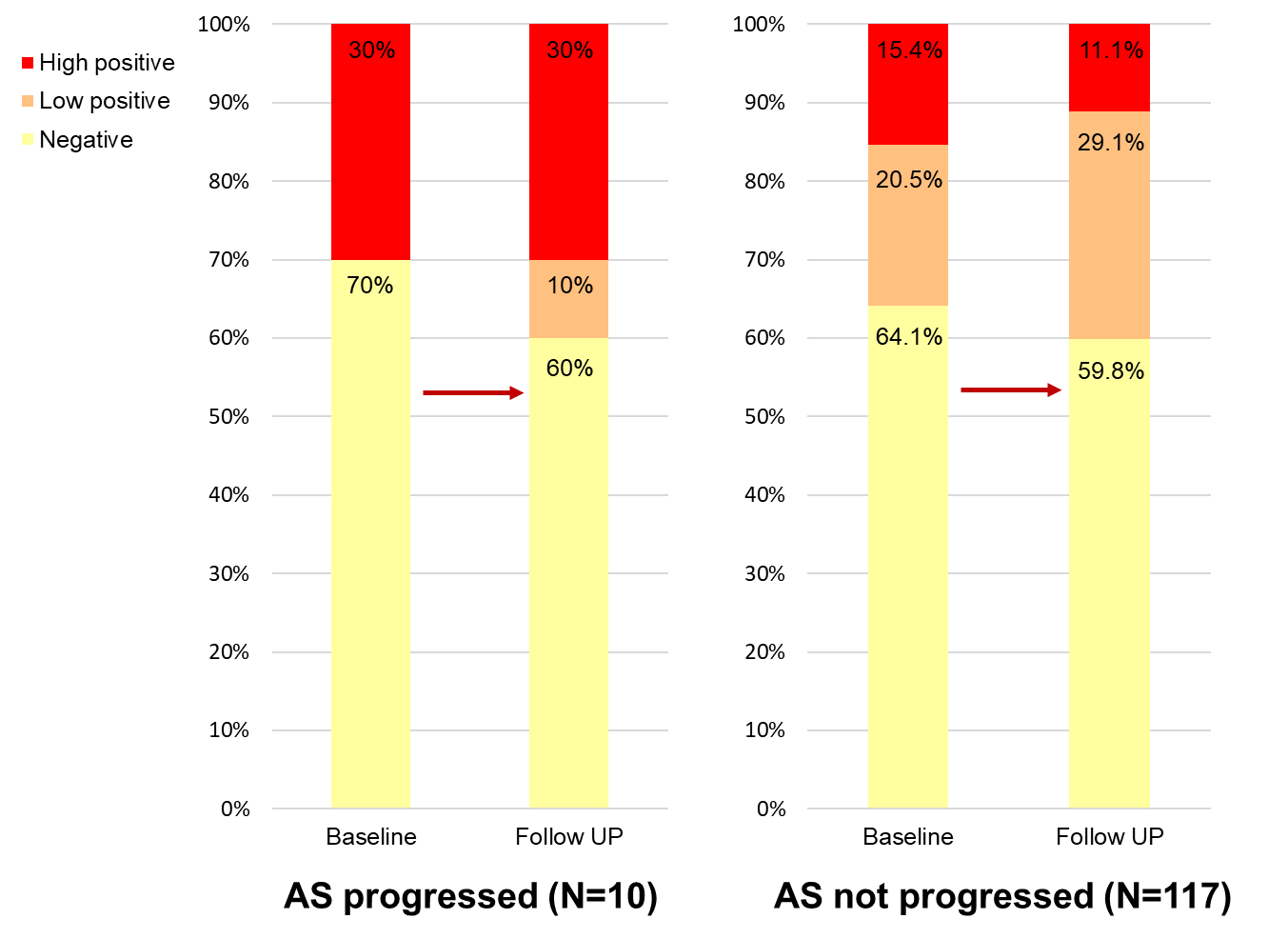


**Supplementary Figure 8. Clinical outcomes according to the combined ILD or RA and anti-CCP antibody titre**

**Supplementary Figure 9. The expression of PAD2 gene in aortic valve interstitial cells**


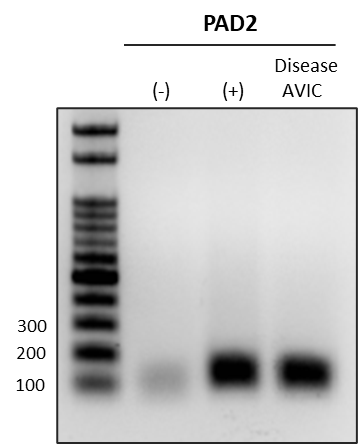


The PCR results confirmed the expression of the PAD2 gene using cDNA extracted from aortic valve tissue of a patient who underwent aortic valve replacement surgery. (-) represents the negative control, which was conducted by excluding the template during the PCR reaction. (+) represents the positive control, which was conducted using MCF7 cells in which the expression of PAD2 was already confirmed. The expected size of the PCR product was 103bp.

**Supplementary Figure 10. Western blot using specific antibodies of citrullinated proteins**

The representative Western blot images to detect the citrullinated fibrinogen, citrullinated vimentin, citrullinated a-enolase using the proteins which were directly extracted from aortic valve tissues.
